# Quality of life in Brazilian medical students: a systematic review and meta-analysis

**DOI:** 10.47626/2237-6089-2022-0497

**Published:** 2024-01-08

**Authors:** Carolina Kakiuthi Martins, Jonas Carneiro Cruz, Renata Dellalibera-Joviliano

**Affiliations:** 1 Faculdade de Medicina de Ribeirão Preto USP Ribeirão Preto SP Brazil Faculdade de Medicina de Ribeirão Preto , Universidade de São Paulo ( USP ), Ribeirão Preto , SP , Brazil .; 2 USP Ribeirão Preto SP Brazil USP , Ribeirão Preto , SP , Brazil .; 3 Departamento de Ciências Biomédicas e da Saúde Universidade do Estado de Minas Gerais Passos MG Brazil Departamento de Ciências Biomédicas e da Saúde , Universidade do Estado de Minas Gerais , Campus Passos, Passos , MG , Brazil .

**Keywords:** Medical students, quality of life, meta-analysis

## Abstract

**Objectives:**

Medical training negatively impacts the quality of life of students. Assessing the well-being of medical students could guide academic policies and future research for improving the mental and physical health status of the population at risk. This study aimed to identify the influence of medical training on the quality of life of Brazilian medical students.

**Methods:**

A systematic review and meta-analysis was conducted according to the Cochrane criteria and reported in accordance with the Preferred Reporting Items for Systematic Reviews and Meta-Analyses protocol (PRISMA). The search was performed by two independent investigators using a predefined protocol registered on the PROSPERO database (CRD42021237926). Data were extracted from PubMed, Embase, and Biblioteca Virtual de Saúde (BVS). For quantitative synthesis, a meta-analysis was conducted to assess the mean difference in the quality of life between medical students at different stages in the academic cycle, stratified by sex. All data were analyzed using the random-effects model, with a confidence interval of 95% (95%CI).

**Results:**

After evaluating the eligibility criteria, five studies were included in the meta-analysis. The data revealed that students in the pre-clinical cycle of the course exhibited higher quality of life scores in the physical (3.05 [1.48-4.62], p < 0.0001) and psychological (3.05 [0.80-5.30], p < 0.0001) domains than students in the clerkship cycle. No statistically significant differences were observed in the environmental (0.78 [-2.92-4.49], p = 0.68) or social domains (1.41 [-0.52-3.34], p = 0.15).

**Conclusion:**

Our analysis revealed that the medical course is associated with decreased quality of life of medical students in the physical and psychological domains. This finding was observed in both men and women. However, these findings should be interpreted with caution given the study limitations. Registration number PROSPERO, CRD42021237926.

## Introduction

The World Health Organization (WHO) has defined quality of life (QoL) as “the individual’s perception of their position in life in the context of the culture and value systems in which they live, and in relation to their goals, expectations, standards, and concerns.” ^1^ It is a broad term that is used to describe an individual’s health, making this assessment more comprehensive. ^2 , 3^

Many observational studies have been conducted worldwide to assess the QoL of medical students using various indices. These studies showed that future physicians were more likely to have poorer scores on QoL, anxiety, and depression indices. ^4 - 6^

Medical students have stressful and overloaded academic lives. The constant proximity to diseases, exhausting medical curriculum, and highly competitive environment appear to negatively impact the students’ well-being. A recent study conducted by Pagnin et al. ^7^ showed that Brazilian medical students exhibited lower QoL scores in psychological and social domains than the general young population. Similar results were found for medical students in New Zealand, ^8^ Italy, ^9^ and South Korea. ^10^ In addition, some of these studies showed that gender has a considerable effect on the QoL of students, but its exact influence is not clear. ^7 , 9^

In this context, assessing the well-being of students could guide academic policies for improving the mental and physical health status of the population at risk. ^11^ Therefore, this systematic review and meta-analysis aimed to investigate the available evidence on the influence of academic training on the well-being of Brazilian medical students. We stratified our analysis by sex to obtain more accurate and reliable results. This strategy was also important for controlling the analysis for this potential confounder and reducing the sources of heterogeneity.

## Materials and methods

This study is reported according to the Preferred Reporting Items for Systematic Reviews and Meta-Analyses (PRISMA) guidelines ^12^ and was designed according to the Cochrane ^13^ criteria for systematic review and meta-analysis protocols. The PRISMA-P checklist for reporting this systematic review ^14^ is presented in Supplementary Material S1.

### Eligibility criteria

The search strategy for this systematic review was to find answers to the following guiding questions structured according to the generic Patient, Exposure, Control, Outcome (PECO) framework.

1. Population: Brazilian medical students in the last academic cycle (5th and 6th years)
2. Exposure: medical training
3. Comparison: Brazilian medical students in the first academic cycle (1st and 2nd years)
4. Outcomes: QoL evaluated using the abbreviated World Health Organization Quality of Life (WHOQOL-Bref) questionnaire assessing four domains: physical, social, psychological, and environmental.

The inclusion criteria were as follows: 1) studies only comparing medical students; 2) studies only comparing students in the first and last academic cycles; 3) studies using the WHOQOL-Bref questionnaire; and 4) studies that included indices stratified by sex for assessing QoL. No restrictions were applied to year of publication, study design, or location.

The exclusion criteria included: 1) studies involving students studying disciplines other than medicine; 2) studies comparing medical interns; 3) studies that did not involve any comparator; 4) studies that did not use the WHOQOL questionnaire as the instrument to assess QoL; 5) studies that were not peer-reviewed; 6) studies conducted outside Brazil; 7) studies with incomplete or missing data (mean and standard deviation [SD]); 8) reviews, letters to the editors, conference summaries, or expert opinions; and 9) studies that assessed QoL using indices not stratified by sex.

### Information sources and search strategy

The PubMed and Embase databases were searched for articles in English. Additionally, Biblioteca Virtual de Saúde (BVS) was searched for articles in Portuguese. The BVS is a space for the integration of health information sources (mostly in Portuguese and Spanish) comprising Literatura Latino-Americana e do Caribe em Ciências da Saúde (LILACS); Index Psicologia – Periódicos; and Banco de Dados em Enfermagem (BDENF). Word combinations and truncations were specifically designed and tailored for each electronic database. The reference list of a previous meta-analysis conducted by Solis and Lotufo-Neto ^2^ was also explored. The complete search strategy used in the databases is presented in Supplementary Table S1. The investigation was conducted on March 1, 2021. No effort was made to contact a subject-matter expert for recommendation of any pertinent articles that could be included.

### Selection process

All articles extracted from the databases were imported into an EndNote library as bibliographic citation files. Endnote was used to manage citations and identify duplicates. All titles, abstracts, and full texts extracted from the databases were screened by two independent reviewers (JCC and CKM). The full texts of potentially relevant studies were assessed. Reasons for excluding studies from the meta-analysis were divided into four categories: (i) lack of data (SD or the total number of participants in each sex group); (ii) studies that did not compare the influence of academic cycles on QoL; (iii) studies that did not use the WHOQOL-Bref as the QoL measurement instrument (including studies using the 36-Item Short Form Survey [SF-36] and the Vida de Estudante e Residente na Área de Saúde [VERAS-Q] questionnaires); and (iv) studies that did not compare the first and last academic cycles. Only articles that met all the eligibility criteria were included in the meta-analysis. All excluded studies are appraised in Supplementary Table S2 (with reasons for exclusion). Any disagreements regarding study inclusion were resolved by consensus between the investigators.

### Data collection process and data items

The data extracted were input to an Excel spreadsheet (Excel, Microsoft, Washington, USA) by one reviewer (JCC). All data were verified by the second reviewer (CKM). Any conflicts in data were verified again and resolved by consensus between the investigators. The following information was extracted from the studies selected for the review: authorship, data collection date, location of study, name of university, type of university (public or private), population (number of students enrolled), proportion of the population analyzed, instrument used to assess QoL, parameters investigated, and predictive factors. No efforts were made to contact the authors regarding incomplete or missing data.

The mean and SD of the WHOQOL scores were obtained for all four domains (physical, social, psychological, and environmental). When results in the studies were provided in the form of crude scores (without transformation into percentages), they were converted to a normalized scale according to the WHO guidelines.

The WHOQOL-Bref is the questionnaire most widely used for research purposes. This tool is a valid and reliable short version of the WHOQOL-100 version. The WHOQOL-Bref comprises 26 questions divided into four domains (physical, psychological, social, and environmental). This instrument was developed to measure individuals’ perceptions of all facets of their QoL. ^15^ A version translated into Brazilian Portuguese was developed in 2000. ^16^

According to Brazilian medical training guidelines, the academic curriculum is divided into three cycles: pre-clinical, clinical, and clerkship. When the studies presented QoL scores by academic year, the first 2 years were defined as the pre-clinical cycle, the 3rd and 4th years as the clinical cycle, and the last 2 years as the clerkship cycle.

Clerkships are regulated by the Ministry of Education (Brazil) through the National Curricular Guidelines (Diretrizes Curriculares Nacionais) for medical courses. This cycle has a minimum duration of 2,700 hours and is performed under professional supervision. The mandatory curriculum includes activities at primary, secondary, and tertiary care levels in the following areas: internal medicine, surgery, obstetrics-gynecology, pediatrics, and public health. ^17^

### Assessment of quality and risk of bias for the studies included in the meta-analysis

The quality of the studies was appraised using the Joanna Briggs Institute’s Critical Appraisal Tools in eight domains. The study domains were classified (yes or no) by two independent reviewers (JCC and CKM) to determine the extent to which a study addressed the possibility of bias in its design. Any disagreements were resolved by consensus. No contact was made with the authors of the studies regarding any unclear information. Publication bias was not assessed because of the small number of studies included.

### Effect measures and quantitative synthesis

The primary outcome was the mean difference in QoL between Brazilian medical students in the last academic cycle (clerkship) and those in the first cycle (pre-clinical) of medical training. Forest plots were created using Review Manager 5.2.7 software (Cochrane Collaboration, Oxford, United Kingdom). The means and SDs of the WHOQOL scores were obtained for four domains (physical, social, psychological, and environmental). Data were stratified by sex. This strategy was used to reduce potential heterogeneity across studies, since women’s baseline QoL scores might have been considerably lower. Weighted mean difference was calculated using generic inverse variance in the random-effects model. Heterogeneity in the subgroups was estimated using the inconsistency index (I ^2^ ) statistical test. Sensitivity analysis was not conducted.

### Certainty assessment

The Grading of Recommendations, Assessment, Development, and Evaluation (GRADE) ^18^ system was used to evaluate the quality of evidence. The quality of evidence was classified into four levels (high, moderate, low, and very low) according to the following parameters: risk of bias, inconsistency, indirectness, imprecision, publication bias, magnitude of effect, dose-response gradient, and residual confounding. This assessment was performed by two independent investigators (JCC and CKM). Any disagreements were resolved by consensus.

## Results

### Study selection

**Figure 1:**
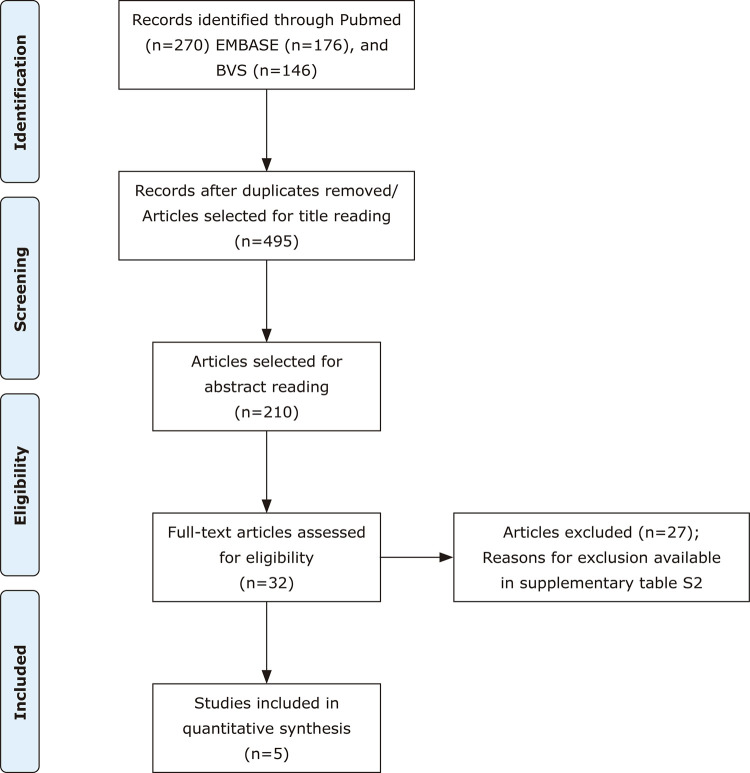
Preferred Reporting Items for Systematic Reviews and Meta-Analyses (PRISMA) flow chart for the summarized results.

We identified 592 articles through our search strategy, 97 of which were duplicates. After evaluating the titles and abstracts, the full texts of 32 studies were assessed. Finally, for quantitative synthesis, five studies (including 1,819 students) were included in the meta-analysis. Figure 1 illustrates the PRISMA flowchart for the summarized results. Supplementary Table S2 shows the detailed characteristics of the studies not included in the meta-analysis, including the reasons for exclusion. The main characteristics of each study are summarized in Table 1 .

**Table 1:** Characteristics of the studies included in the meta-analysis

Author	Data collection date	Location	University (abbreviation) (type of institution)	Population (n)	Percentage of population enrolled	Comparisons	Negative predictor factors
Alves ^19^	Aug 2006-Apr 2007	Recife (PE)	Multicenter: UFPE (public), UPE (public), EPM) (private).	First and last semester students (370)	83.2 (1st year) 90.9 (last year)	1st and last semester	Last semester
Pagnin ^7^	Not informed	Niterói (RJ)	UFF (public)	2nd, 4th, and 6th year students (206)	86.4	Academic year, gender, young general population	Female sex, being a medical student
Serinolli ^22^	Oct-Nov 2014	São Paulo (SP)	UNINOVE (private)	All students (405)	65.3	Academic year, body mass index, gender, daily traveling time, housing conditions, parents’ educational background, religiosity, smoking	Absence of religious beliefs, high body mass index, female sex, long traveling time, lack of a physician in the family
Cazolari ^20^	Aug-Oct 2017	São Paulo (SP)	UNIFESP (public)	All students (302)	42.0	Academic cycle, burnout, gender	Last cycle
Paro ^21^	Aug 2011-Aug 2012	Multicenter (BA, SP, PB, PR, RO, PI, CE, RS, GO, MS, RJ, TO, MG)	Multicenter (10 public, 12 private medical schools)	All students (1,350)	81.8	Academic cycle, empathy, gender	Female sex, low resilience, low education environment, low levels of physical activity
Tempski ^4^						Anxiety, burnout, depression, educational environment, resilience,	
Enns ^23^						Educational environment	
Peleias ^24^						Leisure time physical activity	

EPM = Escola Pernambucana de Medicina; UFF = Universidade Federal Fluminense; UFPE = Universidade Federal de Pernambuco; UNIFESP = Universidade Federal de São Paulo; UNINOVE = Universidade Nove de Julho; UPE = Universidade Estadual de Pernambuco.

### Study characteristics

We included five studies ^7 , 19 - 22^ with a total of 1,819 students. The studies collected data from February 2006 ^19^ to October 2017. ^20^ The study locations included all five macroregions of Brazil (South, North, Northeast, Southeast, and Midwest). A single multicenter study ^21^ evaluated medical students in 13 of 27 states, including Bahia (BA), São Paulo (SP), Paraíba (PB), Paraná (PR), Rondônia (RO), Rio Grande do Sul (RS), Piauí (PI), Ceará (CE), Goiás (GO), Mato Grosso do Sul (MS), Rio de Janeiro (RJ), Tocantins (TO), and Minas Gerais (MG). Other single-center studies were performed in three states: Pernanbuco (PE), ^19^ SP, ^20 , 22^ and RJ. ^7^ Thirteen states were not included in any study: Espírito Santo (ES), Mato Grosso (MT), Acre (AC), Sergipe (SE), Paraíba (PB), Rio Grande do Norte (RN), Maranhão (MA), Pará (PA), Amapá (AP), Roraima (RR), Amazonas (AM), Santa Catarina (SC), and Alagoas (AL). In contrast, SP (the most populous state in Brazil) was the most common research location.

Other parameters investigated included sex, lack of a physician in the family, low educational environment, absence of religious beliefs, lack of physical activity, burnout, difficulty sleeping, stress, body mass index, presence of comorbidities, low resilience, low levels of physical activity, depression, and anxiety. Thirteen of the institutions evaluated were public and 15 were private. The percentage of students enrolled on each study ranged from 42% ^20^ to 90.9% ^19^ ( Table 1 ).

### Synthesis of results

**Figure 2:**
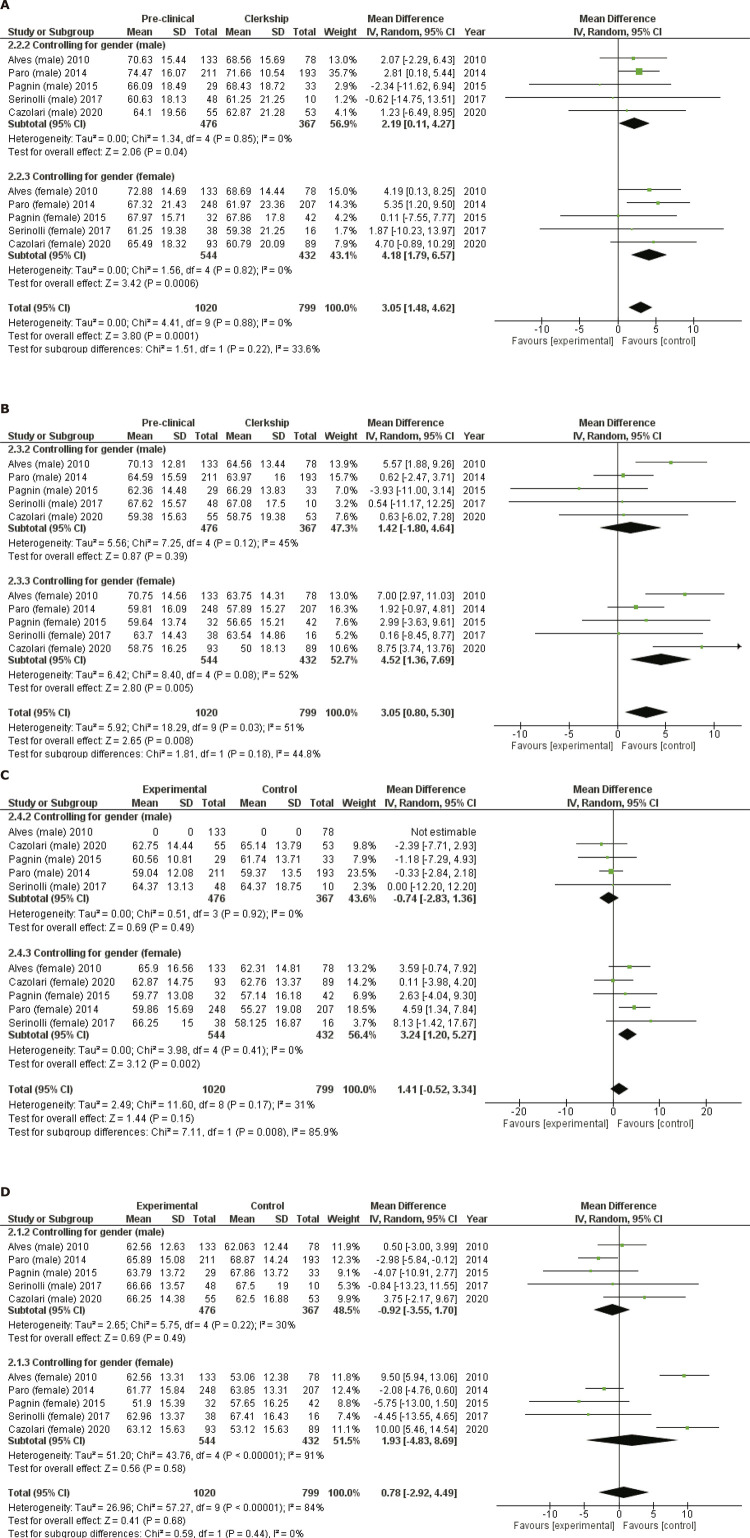
Forest plots (stratified by sex) for the difference in the quality of life (QoL) scores of Brazilian medical students in the social (A), psychological (B), environmental (C), and physical (D) domains. 95%CI = 95% confidence interval; df = degrees of freedom; I 2 = inconsistency index; SD = standard deviation.

The meta-analysis revealed that medical students in the last academic cycle had lower QoL scores in the social and psychological domains than those in the first academic cycle. The mean difference in the social domain scores was 2.19 (0.11-4.27) (p = 0.04) for men, 4.18 (1.79-6.57) (p = 0.006) for women, and 3.05 (1.48-4.62) (p < 0.0001) for the overall effect (Figure 2A). The heterogeneity of this outcome was considerably low for both subsets and the overall effect (Tau ^2^ = 0.00; chi ^2^ = 4.41, degrees of freedom [df] = 9, p = 0.88; I ^2^ = 0%).

The mean difference in the psychological domain scores was 4.52 (1.36-7.69) (p = 0.005) for women and 3.05 (0.80-5.30) (p = 0.008) for the overall effect. No statistically significant difference was observed when only men were analyzed: 1.42 (-1.80-4.64) (p = 0.39). Moderate heterogeneity was identified for men (Tau ^2^ = 5.56; chi ^2^ = 7.25, df = 4, p = 0.12; I ^2^ = 45%), women (Tau ^2^ = 6.42; chi ^2^ = 8.40, df = 4, p = 0.08; I ^2^ = 52%), and the overall effect (Tau ^2^ = 5.92; chi ^2^ = 18.29, df = 9, p = 0.03; I ^2^ = 51%) (Figure 2B).

The mean difference in the environmental domain scores was 3.24 (1.20-5.27) (p = 0.002) for women. However, no statistically significant difference was observed in the scores for men (-0.74 [-2.83-1.36]; p = 0.49) or the overall effect (1.41 [-0.52-3.34]; p = 0.15). Heterogeneity was considerably low for the subset analyses (I ^2^ = 0) but remained at a moderate level for the overall estimate (Tau ^2^ = 2.49; chi ^2^ = 11.60, df = 8, p = 0.17; I ^2^ = 31%) (Figure 2C).

Finally, no difference was observed in the physical domain scores for men (-0.92 [-3.55-1.70], p = 0.22), women (1.93 [-4.83-8.69], p = 0.58), or the overall estimate (0.78 [-2.92-4.49] p = 0.68). Although heterogeneity was moderate in men (Tau ^2^ = 2.65; chi ^2^ = 5.75, df = 4, p = 0.22; I ^2^ = 30%), it was high for women (Tau ^2^ = 51.20; chi ^2^ = 43.76, df = 4, p < 0.00001; I ^2^ = 91%) and in the overall estimate (Tau ^2^ = 26.96; chi ^2^ = 57.27, df = 9, p < 0.00001; I ^2^ = 84%) (Figure 2D).

### Risk of bias

Overall, all articles included in the meta-analysis presented a moderate risk of bias. All studies had a low risk of bias in terms of exposure to medical training and the application of statistical analyses. The studies conducted by Paro et al., ^21^ Alves et al., ^19^ and Pagnin et al. ^7^ included a large sample of enrolled students and had a low risk of bias in terms of participant selection. In contrast, the studies by Serinolli and Novaretti ^22^ and Cazolari et al. ^20^ had a moderate risk of bias. In addition, all studies were highly biased in terms of assessing the outcome, because questionnaires are subjective forms of evaluation ( Table 2 ).

**Table 2:** Risk of bias of the included studies

Reference	Were the criteria for inclusion in the sample clearly defined?	Were the study subjects and the setting described in detail?	Was the exposure measured in a valid and reliable way?	Were objective, standard criteria used for measurement of the condition?	Were confounding factors identified?	Were strategies to deal with confounding factors stated?	Were the outcomes measured in a valid and reliable way?	Was appropriate statistical analysis used?
Alves ^19^	Yes	Yes	Yes	Yes	No	No	No	Yes
Pagnin ^7^	Yes	Yes	Yes	Yes	No	No	No	Yes
Serinolli ^22^	Yes	Yes	Yes	Yes	Yes	Yes	No	Yes
Cazolari ^20^	Yes	Yes	Yes	Yes	No	No	No	Yes
Paro ^21^	Yes	Yes	Yes	Yes	Yes	Yes	No	Yes

### Confidence in cumulative evidence

According to the GRADE tool, the overall quality of evidence was very low. Methodological limitations (lack of randomization), inconsistency (lack of control for confounding factors such as economic class and ethnicity), and inaccuracy (subjectivity of questionnaires) were the main factors responsible for lowering the evidence level. In addition, the magnitude of the effect was not substantial.

## Discussion

This systematic review and meta-analysis investigated the available evidence on reduction sin the QoL of Brazilian medical students during their undergraduate studies. Based on the available literature and eligibility criteria, our data revealed that medical training was a negative predictor of students’ well-being. The meta-analysis revealed that medical students in the clerkship cycle (5th and 6th years) had an average of 3.05% lower QoL scores in both psychological and social domains than students in the pre-clinical cycle.

In part, these results might be because of the stressful and competitive environment faced by medical students. In this context, the overwhelming burden of educational information, extensive workload, and the demand for high academic performance can lead to development of mental disorders such as burnout, anxiety, and depression. ^11 , 25^ In addition, other causes of student distress include personal events, ethical conflicts, exposure to human death and suffering, student abuse (verbal, physical, or sexual), and educational debt. Although these sources of stress may vary widely across training years, they tend to become more intense by the end of medical training. ^11^

Although the heterogeneity (I ^2^ ) scores for the environmental and social domains reduced to 0% after controlling for sex, those in the physical and psychological domains remained moderate. However, the variations in analyses could be because of the study designs and the academic curriculum. In this regard, some studies categorized their data by years of medical training (first to sixth), while others stratified their results according to academic cycle (pre-clinical, clinical, and clerkship). In addition, curricular differences between courses, especially problem-based learning (PBL) and lecture-based learning (LBL), are a potential source of heterogeneity. Some Brazilian medical schools employ a PBL methodology based on the application of acquired knowledge and integration of new information. ^26^ In contrast, other universities use the traditional LBL approach, in which the students adopt the role of a passive learner.

Although not investigated in this review, the difference in the QoL between genders could be explained by sex differences in socioeconomic status. ^27 , 28^ In this context, gender disparities appear to have a significant effect on the well-being of women. ^29^ Women are more likely to suffer from mental disorders (such as depression, anxiety, or stress), ^30^ while men experience social issues such as substance abuse. ^31^ Additionally, men are more physically active, ^32^ less prone to suffer from chronic pain, ^33^ and have higher self-esteem ^34^ than women. In this scenario, mental status could be a confounder, since recent studies have shown that female medical students exhibited higher risk of developing mental disorders, such as depression and anxiety, than male students. ^35 , 36^

### Comparison with other studies

Although the influence of medical training on the QoL of Brazilian medical students has already been investigated by Solis and Lotufo-Neto ^2^ in a previous meta-analysis, they presented their results in terms of effect sizes (Hedges g). This approach can lead to spurious results and the magnitude of effects is difficult to interpret, since the results are presented as SD. ^37^ In contrast, our analysis was performed using weighted mean difference. This strategy yields an objective estimate (expressed in percentages), which is easier for clinicians and researchers to understand.

Nevertheless, the results of our analysis corroborate with those of the previous meta-analysis conducted by Solis and Lotufo-Neto, ^2^ which revealed that medical training is associated with reduced QoL scores in Brazilian students. However, our data provide more accurate results as we performed the meta-analysis after stratification by sex. This variable may act as a confounder, explaining some part of the heterogeneity between the overall estimates.

We also found similarities between our results and those of two cross-sectional studies conducted in China ^38^ and Saudi Arabia. ^39^ Both of those studies showed lower QoL scores in the psychological and social domains in clerkship students than in students in the pre-clinical cycle.

### Limitations

There are significant limitations to our data. First, QoL assessment using questionnaires is based on subjective interpretations, which each respondent perceives and expresses differently. In addition, it is difficult to control for potential confounding factors in observational studies and our analysis did not allow the insertion of adjusted scores. Further, sociodemographic variables of different medical studies could be different, which could affect the results and cause problems in interpreting the results. Although a better strategy would be to include longitudinal studies, only Moutinho et al., ^40^ utilized this study design. Moreover, they only conducted 2 years of follow-up.

We also found that the number of clerkship students enrolled was lower than the number of students in the first academic cycle. However, this finding could be because of the academic routine, which makes it challenging to obtain responses to questionnaires from these students.

In addition, not all medical students were included in the meta-analysis because of differences in the instruments used to asses QoL. We identified studies that used the VERAS-Q and the SF-36 questionnaires for data collection. The SF-36 is being increasingly used in the scientific literature, but its validity as a measure of overall QoL is questionable. ^41^ The VERAS-Q is a specific questionnaire developed in Brazil to appraise the QoL of medical students. It contains 90 items with a Likert-scale response format. This tool was validated in 2009 with 800 medical students from 75 Brazilian medical schools. However, this instrument has not been widely adopted or approved worldwide. ^42^

Although these questionnaires are valid instruments for assessing QoL, they are not interchangeable. Therefore, mixing the constructs could have significantly increased the sources of heterogeneity. ^43^

Many of the studies assessed in full-text form during the data extraction process did not control for sex differences or provided insufficient data. Therefore, the differences in the QoL across the course cycles could have been directly affected by imbalances between numbers of male and female participants in the studies. These articles were not therefore included in the final synthesis.

### Future directions

Although several studies have been conducted to estimate the impact of medical training on the QoL of Brazilian medical students, most studies concerning these outcomes were not well replicated, did not control for potential confounders, or used low-quality/nuclear-quality measurement instruments. We therefore recommend that studies should employ the WHOQOL-Bref questionnaire in future investigations.

We strongly recommend that studies that determine the QoL index should control for sex. This is possible by stratifying the data according to sex (reporting SD for each subgroup) or performing covariance analyses including sex as a covariable.

Future research should address the influence of mental disorders, economic status, ethnicity, and social class on student well-being. These variables could act as confounders, explaining the differences in QoL scores observed in other studies. Further studies should focus on whether the PBL or LBL academic curriculum influences student well-being. Furthermore, since affirmative action policies have widely changed in Brazil in the last decade, new studies should be conducted to investigate the impact of medical training on the QoL of this specific class of students.

### Contributions of this study

We believe that these findings will be a valuable tool to guide future research and design specific academic policies for improving the well-being of particular students. Therefore, we recommend that the psychological health of medical students should be monitored and support should be provided within the educational institution. In addition, we maintain that workload could be revised.

## Supplementary Material


