# Supplementary material for: Quality of life in Brazilian medical students: a systematic review and meta-analysis
Source: Trends Psychiatry Psychother. 2024 Jan 8;46:e20220497. doi: 10.47626/2237-6089-2022-0497 (PMC11140770; doi:10.47626/2237-6089-2022-0497)
Supplement: Supplementary file 1 [file 2238-0019-trends-46-e20220497-suppl.pdf]

http://doi.org/10.47626/2237-6089-2022-0497

Supplementary Material S1

PRISMA 2020 expanded checklist

Note: This expanded checklist details elements recommended for reporting for each PRISMA 2020 item. Non-italicized elements are considered ‘essential’ and should be reported in the main report or as supplementary material for all systematic reviews (except for those preceded by “If…”, which should only be reported where applicable). Elements written in italics are ‘additional’, and while not essential, provide supplementary information that may enhance the completeness and usability of systematic review reports. Note that elements presented here are an abridged version of those presented in the explanation and elaboration paper, with references and some examples removed. Consulting the explanation and elaboration paper is recommended if further clarity or information is required (doi:10.1136/bmj.n160).

Section and Topic	Item #	Elements recommended for reporting	
TITLE			
TITLE	1	• Identify the report as a systematic review in the title.<br>• Report an informative title that provides key information about the main objective or question the review addresses (e.g. the population(s) and intervention(s) the review addresses).<br>• Consider providing additional information in the title, such as the method of analysis used, the designs of included studies, or an indication that the review is an update of an existing review, or a continually updated (“living”) systematic review.	• Quality of life in Brazilian medical students: A systematic review and meta-analysis<br>•
ABSTRACT			
ABSTRACT	2	• Report an abstract addressing each item in the PRISMA 2020 for Abstracts checklist.	• See abstract
INTRODUCTION			
RATIONALE	3	• Describe the current state of knowledge and its uncertainties.<br>• Articulate why it is important to do the review.<br>• If other systematic reviews addressing the same (or a largely similar) question are available, explain why the current review was considered necessary. If the review is an update or replication of a particular systematic review, indicate this and cite the previous review.<br>• If the review examines the effects of interventions, also briefly describe how the intervention(s) examined might work.<br>• If there is complexity in the intervention or context of its delivery (or both) (e.g. multi-component interventions, equity considerations), consider presenting a logic model to visually display the hypothesised relationship between intervention components and outcomes.	• Introduction paragraph 1 and 2<br>• Introduction paragraph 3<br>• Introduction paragraph 4<br>• Not applicable<br>• Not applicable
OBJECTIVES	4	• Provide an explicit statement of all objective(s) or question(s) the review addresses, expressed in terms of a relevant question formulation framework.<br>• If the purpose is to evaluate the effects of interventions, use the Population, Intervention, Comparator, Outcome (PICO) framework or one of its variants, to state the comparisons that will be made.	Introduction “Therefore, this systematic review and meta-analysis aimed to investigate the available evidence on the the influence of academic training in the Brazilian medical student’s well-being.<br>• “Not applicable

METHODS			
ELIGIBILITY CRITERIA	5	• Specify all study characteristics used to decide whether a study was eligible for inclusion in the review, that is, components described in the PICO framework or one of its variants, and other characteristics, such as eligible study design(s) and setting(s), and minimum duration of follow-up.<br>• Specify eligibility criteria with regard to report characteristics, such as year of dissemination, language, and report status (e.g. whether reports, such as unpublished manuscripts and conference abstracts, were eligible for inclusion).<br>• Clearly indicate if studies were ineligible because the outcomes of interest were not measured, or ineligible because the results for the outcome of interest were not reported.<br>• Specify any groups used in the synthesis (e.g. intervention, outcome and population groups) and link these to the comparisons specified in the objectives (item #4).<br>• Consider providing rationales for any notable restrictions to study eligibility.	• 1. Population: Brazilian medical students in the last academic cycle (5th and 6th year), 2. Intervention: Medical training, 3. Comparison: Brazilian medical students in the first academic cycle (1st and 2nd year), 4. Outcomes: Quality of life evaluated under the WHOQOL questionnaire in four domains: physical, social, psychological, and environment<br>• No restriction study's year was applied, inclusion: Studies conducted in Portuguese and English. Exclusion: Studies that did not follow the peer review mechanism;<br>• Exclusion: articles with incomplete or missing data (mean and standard deviation)<br>• Not applicable<br>• Randomization is not possible for this exposure
INFORMATION SOURCES	6	• Specify the date when each source (e.g. database, register, website, organisation) was last searched or consulted.<br>• If bibliographic databases were searched, specify for each database its name (e.g. MEDLINE, CINAHL), the interface or platform through which the database was searched (e.g. Ovid, EBSCOhost), and the dates of coverage (where this information is provided).<br>• If study registers, regulatory databases and other online repositories were searched, specify the name of each source and any date restrictions that were applied.	• . The investigation was carried out from March 1, 2021, to August 1, 2021.<br>• The following databases were searched for articles in English: PUBMED and EMBASE. An additional investigation was conducted in the Biblioteca Virtual de Saúde (BVS) for articles in Portuguese.<br>• Not applicable
Section and Topic	Item #	Elements recommended for reporting	
		• If websites, search engines or other online sources were browsed or searched, specify the name and URL of each source.<br>• If organisations or manufacturers were contacted to identify studies, specify the name of each source.<br>• If individuals were contacted to identify studies, specify the types of individuals contacted (e.g. authors of studies included in the review or researchers with expertise in the area).<br>• If reference lists were examined, specify the types of references examined (e.g. references cited in study reports included in the systematic review, or references cited in systematic review reports on the same or similar topic).<br>• If cited or citing reference searches (also called backward and forward citation searching) were conducted, specify the bibliographic details of the reports to which citation searching was applied, the citation index or platform used (e.g. Web of Science), and the date the citation searching was done.<br>• If journals or conference proceedings were consulted, specify of the names of each source, the dates covered and how they were searched (e.g. handsearching or browsing online).	• -.<br>• Not applicable<br>• No efforts were made<br>• The reference list of the previous meta-analysis conducted by Solis was also explored<br>• Not applicable<br>• Not applicable

SEARCH STRATEGY	7	• Provide the full line by line search strategy as run in each database with a sophisticated interface (such as Ovid), or the sequence of terms that were used to search simpler interfaces, such as search engines or websites.<br>• Describe any limits applied to the search strategy (e.g. date or language) and justify these by linking back to the review's eligibility criteria.<br>• If published approaches, including search filters designed to retrieve specific types of records or search strategies from other systematic reviews, were used, cite them. If published approaches were adapted, for example if search filters are amended, note the changes made.<br>• If natural language processing or text frequency analysis tools were used to identify or refine keywords, synonyms or subject indexing terms to use in the search strategy, specify the tool(s) used.<br>• If a tool was used to automatically translate search strings for one database to another, specify the tool used.<br>• If the search strategy was validated, for example by evaluating whether it could identify a set of clearly eligible studies, report the validation process used and specify which studies were included in the validation set.<br>• If the search strategy was peer reviewed, report the peer review process used and specify any tool used such as the Peer Review of Electronic Search Strategies (PRESS) checklist.<br>• If the search strategy structure adopted was not based on a PICO-style approach, describe the final conceptual structure and any explorations that were undertaken to achieve it.	• . The complete search strategy carried out in the databases are presented in supplementary file 2<br>• Inclusion: Studies conducted in Portuguese and English.<br>• Not applicable<br>• Not applicable<br>• Not applicable<br>• Not applicable<br>• Not applicable<br>•
SELECTION PROCESS	8	Recommendations for reporting regardless of the selection processes used:<br>• Report how many reviewers screened each record (title/abstract) and each report retrieved, whether multiple reviewers worked independently at each stage of screening or not, and any processes used to resolve disagreements between screeners.<br>• Report any processes used to obtain or confirm relevant information from study investigators.<br>• If abstracts or articles required translation into another language to determine their eligibility, report how these were translated.<br>Recommendations for reporting in systematic reviews using automation tools in the selection process:<br>• Report how automation tools were integrated within the overall study selection process.<br>• If an externally derived machine learning classifier was applied (e.g. Cochrane RCT Classifier), either to eliminate records or to replace a single screener, include a reference or URL to the version used. If the classifier was used to eliminate records before screening, report the number eliminated in the PRISMA flow diagram as 'Records marked as ineligible by automation tools'.<br>• If an internally derived machine learning classifier was used to assist with the screening process, identify the software/classifier and version, describe how it was used (e.g. to remove records or replace a single screener) and trained (if relevant), and what internal or external validation was done to understand the risk of missed studies or incorrect classifications.<br>• If machine learning algorithms were used to prioritise screening (whereby unscreened records are continually re-ordered based on screening decisions), state the software used and provide details of any screening rules applied.<br>Recommendations for reporting in systematic reviews using crowdsourcing or previous 'known' assessments	• All articles extracted from the databases were imported to an Endnote library as bibliographic citation files. The software was also used to manage citations and identify duplicates. All titles, abstracts, and full texts extracted from the databases were screened by two independent reviewers (JCC and CKM). The potentially relevant studies were assessed in the full-text form. Only the articles that met all elibility criteria were included in the meta-analysis.<br>• Not applicable<br>• Not applicable<br>• Not applicable<br>• Not applicable<br>• Not applicable

in the selection process:

Section and Topic	Item #	Elements recommended for reporting	
		• If crowdsourcing was used to screen records, provide details of the platform used and specify how it was integrated within the overall study selection process.<br>• If datasets of already-screened records were used to eliminate records retrieved by the search from further consideration, briefly describe the derivation of these datasets.	• Not applicable<br>• Not applicable
DATA COLLECTION PROCESS	9	• Report how many reviewers collected data from each report, whether multiple reviewers worked independently or not, and any processes used to resolve disagreements between data collectors.<br>• Report any processes used to obtain or confirm relevant data from study investigators.<br>• If any automation tools were used to collect data, report how the tool was used, how the tool was trained, and what internal or external validation was done to understand the risk of incorrect extractions.<br>• If articles required translation into another language to enable data collection, report how these articles were translated.<br>• If any software was used to extract data from figures, specify the software used.<br>• If any decision rules were used to select data from multiple reports corresponding to a study, and any steps were taken to resolve inconsistencies across reports, report the rules and steps used.	• The extracted data were gathered in an orderly fashion in an Excel spreadsheet (Excel, Microsoft, Washington, USA) by one reviewer (JCC). All information was verified by a second reviewer (CKM). Any conflicting information was verified again and solved by consensus between the investigators<br>•). All information was verified by a second reviewer (CKM).<br>• Not applicable<br>• Not applicable<br>• Not applicable<br>• Not applicable

DATA ITEMS (outcomes)	10a	• List and define the outcome domains and time frame of measurement for which data were sought.<br>• Specify whether all results that were compatible with each outcome domain in each study were sought, and if not, what process was used to select results within eligible domains.<br>• If any changes were made to the inclusion or definition of the outcome domains, or to the importance given to them in the review, specify the changes, along with a rationale.<br>• If any changes were made to the processes used to select results within eligible outcome domains, specify the changes, along with a rationale.<br>• Consider specifying which outcome domains were considered the most important for interpreting the review's conclusions and provide rationale for the labelling (e.g. "a recent core outcome set identified the outcomes labelled 'critical' as being the most important to patients").	• The scores and standard deviation of the whoqol questionnaire were assessed in the four domains (physical, social, psychological and environmental).<br>• The academic cycle was divided in three groups accordin Brazilian medical training guidelines: pre-clinical, clinical, and clerkship. When the articles described the quality of life scores by year of study, the first two years were placed in the pre-clinical step, the 3rd and 4th years were categorized as clinical and the last two years as clerkship.<br>• Similar as above<br>• Similar as above<br>• Not applicable
DATA ITEMS (other variables)	10b	• List and define all other variables for which data were sought (e.g. participant and intervention characteristics, funding sources).<br>• Describe any assumptions made about any missing or unclear information from the studies.<br>• If a tool was used to inform which data items to collect, cite the tool used.	• Not applicable<br>• Not applicable<br>• Not applicable
STUDY RISK OF BIAS ASSESSMENT	11	• Specify the tool(s) (and version) used to assess risk of bias in the included studies.<br>• Specify the methodological domains/components/items of the risk of bias tool(s) used.<br>• Report whether an overall risk of bias judgement that summarised across domains/components/items was made, and if so, what rules were used to reach an overall judgement.<br>• If any adaptations to an existing tool to assess risk of bias in studies were made, specify the adaptations.<br>• If a new risk of bias tool was developed for use in the review, describe the content of the tool and make it publicly accessible.<br>• Report how many reviewers assessed risk of bias in each study, whether multiple reviewers worked independently, and any processes used to resolve disagreements between assessors.<br>• Report any processes used to obtain or confirm relevant information from study investigators.<br>• If an automation tool was used to assess risk of bias, report how the automation tool was used, how the tool was trained, and details on the tool's performance and internal validation.	• The quality of the studies was appraised by the Joanna Brigs institute tool<br>• Not applicable<br>• Not applicable<br>• Not applicable<br>• These classifications were made by two independent reviewers (JCC and CKM) and any disagreements were addressed to a third reviewer (CMA).<br>• No contact was made with the authors regarding unclear information.<br>• Not applicable
EFFECT MEASURES	12	• Specify for each outcome (or type of outcome [e.g. binary, continuous]), the effect measure(s) (e.g. risk ratio, mean difference) used in the synthesis or presentation of results.<br>• State any thresholds (or ranges) used to interpret the size of effect (e.g. minimally important difference; ranges for no/trivial, small, moderate and large effects) and the rationale for these thresholds.	• The primary outcome was the mean difference in the quality of life between Brazilian medical students in the last academic cycle (clerkship) compared to those in the begging of the medical trainining (pre-clinical stage).<br>• Not applicable
Section and Topic	Item #	Elements recommended for reporting	
		• If synthesized results were re-expressed to a different effect measure, report the method used to re-express results (e.g. meta-analysing risk ratios and computing an absolute risk reduction based on an assumed comparator risk).	• Not applicable<br>• Not applicable

		• Consider providing justification for the choice of effect measure.	
SYNTHESIS METHODS (eligibility for synthesis)	13a	• Describe the processes used to decide which studies were eligible for each synthesis.	• Not applicable
SYNTHESIS METHODS (preparing for synthesis)	13b	• Report any methods required to prepare the data collected from studies for presentation or synthesis, such as handling of missing summary statistics, or data conversions.	• When the results were given in the crude scores (without transforming into a percentage scale), they were converted in the normalized scale according to the WHO normative.
SYNTHESIS METHODS (tabulation and graphical methods)	13c	• Report chosen tabular structure(s) used to display results of individual studies and syntheses, along with details of the data presented.<br>• Report chosen graphical methods used to visually display results of individual studies and syntheses.<br>• If studies are ordered or grouped within tables or graphs based on study characteristics (e.g. by size of the study effect, year of publication), consider reporting the basis for the chosen ordering/grouping.<br>• If non-standard graphs were used, consider reporting the rationale for selecting the chosen graph.	• **Results:** The main characteristics of each study are summarized in Table 1.<br>• Forest plots were created with the aim of the Review Manager 5.2.7 software (Cochrane Collaboration, Oxford, United Kingdom).<br>• The data was stratified according to sex. This strategy was performed to reduce potential heterogeneity across studies as the baseline QoL index might be considerably different between these groups.
SYNTHESIS METHODS (statistical synthesis methods)	13d	• If statistical synthesis methods were used, reference the software, packages and version numbers used to implement synthesis methods.<br>• If it was not possible to conduct a meta-analysis, describe and justify the synthesis methods or summary approach used.<br>• If meta-analysis was done, specify: o the meta-analysis model (fixed-effect, fixed-effects or random-effects) and provide rationale for the selected model. o the method used (e.g. Mantel-Haenszel, inverse-variance).<br>o any methods used to identify or quantify statistical heterogeneity (e.g. visual inspection of results, a formal statistical test for heterogeneity, heterogeneity variance (τ), inconsistency (e.g. I^2), and prediction intervals).<br>• If a random-effects meta-analysis model was used: o specify the between-study (heterogeneity) variance estimator used (e.g. DerSimonian and Laird, restricted maximum likelihood (REML)).<br>o specify the method used to calculate the confidence interval for the summary effect (e.g. Wald-type confidence interval, Hartung-Knapp-SidikJonkman).<br>o consider specifying other details about the methods used, such as the method for calculating confidence limits for the heterogeneity variance.<br>• If a Bayesian approach to meta-analysis was used, describe the prior distributions about quantities of	• Forest plots were created with the aim of the Review Manager 5.2.7 software (Cochrane Collaboration, Oxford, United Kingdom).<br>• Not applicable<br>• The weighted mean difference was calculated using the generic inverse variance model in the random effects model<br>• Heterogeneity in the sub-groups was estimated by the I^2 statistic test.<br>• I^2<br>• Not applicable<br>• Not applicable<br>• Not applicable<br>• Not applicable

		interest (e.g. intervention effect being analysed, amount of heterogeneity in results across studies).<br>• If multiple effect estimates from a study were included in a meta-analysis, describe the method(s) used to model or account for the statistical dependency (e.g. multivariate meta-analysis, multilevel models or robust variance estimation).<br>• If a planned synthesis was not considered possible or appropriate, report this and the reason for that decision.	
SYNTHESIS METHODS (methods to explore heterogeneity)	13e	• If methods were used to explore possible causes of statistical heterogeneity, specify the method used (e.g. subgroup analysis, meta-regression).<br>• If subgroup analysis or meta-regression was performed, specify for each: o which factors were explored, levels of those factors, and which direction of effect modification was expected and why (where possible).<br>o whether analyses were conducted using study-level variables (i.e. where each study is included in one subgroup only), within-study contrasts (i.e. where data on subsets of participants within a study are available, allowing the study to be included in more than one subgroup), or some combination of the above.<br>o how subgroup effects were compared (e.g. statistical test for interaction for subgroup analyses).<br>• If other methods were used to explore heterogeneity because data were not amenable to meta-analysis of effect estimates (e.g. structuring tables to examine variation in results across studies based on subpopulation), describe the methods used, along with the factors and levels.<br>• If any analyses used to explore heterogeneity were not pre-specified, identify them as such.	• The data was stratified according to sex.<br>• This strategy was performed to reduce potential heterogeneity across studies as the baseline QoL index might be considerably lower in women.<br>• Not applicable<br>• Not applicable<br>• Not applicable<br>• Not applicable
Section and Topic	Item #	Elements recommended for reporting	
SYNTHESIS METHODS (sensitivity analyses)	13f	• If sensitivity analyses were performed, provide details of each analysis (e.g. removal of studies at high risk of bias, use of an alternative meta-analysis model).<br>• If any sensitivity analyses were not pre-specified, identify them as such.	• Not applicable<br>• No sensitivity analysis was conducted

REPORTING BIAS ASSESSMENT	14	• Specify the methods (tool, graphical, statistical or other) used to assess the risk of bias due to missing results in a synthesis (arising from reporting biases).<br>• If risk of bias due to missing results was assessed using an existing tool, specify the methodological components/domains/items of the tool, and the process used to reach a judgement of overall risk of bias.<br>• If any adaptations to an existing tool to assess risk of bias due to missing results were made, specify the adaptations.<br>• If a new tool to assess risk of bias due to missing results was developed for use in the review, describe the content of the tool and make it publicly accessible.<br>• Report how many reviewers assessed risk of bias due to missing results in a synthesis, whether multiple reviewers worked independently, and any processes used to resolve disagreements between assessors.<br>• Report any processes used to obtain or confirm relevant information from study investigators.<br>• If an automation tool was used to assess risk of bias due to missing results, report how the automation tool was used, how the tool was trained, and details on the tool's performance and internal validation.	• Not applicable<br>• Not applicable<br>• Not applicable<br>• Not applicable<br>• Not applicable<br>• Not applicable<br>• Not applicable<br>• Not applicable<br>• Not applicable
CERTAINTY ASSESSMENT	15	• Specify the tool or system (and version) used to assess certainty (or confidence) in the body of evidence.<br>• Report the factors considered (e.g. precision of the effect estimate, consistency of findings across studies) and the criteria used to assess each factor when assessing certainty in the body of evidence.<br>• Describe the decision rules used to arrive at an overall judgement of the level of certainty, together with the intended interpretation (or definition) of each level of certainty.<br>• If applicable, report any review-specific considerations for assessing certainty, such as thresholds used to assess imprecision and ranges of magnitude of effect that might be considered trivial, moderate or large, and the rationale for these thresholds and ranges (item #12).<br>• If any adaptations to an existing tool or system to assess certainty were made, specify the adaptations.<br>• Report how many reviewers assessed certainty in the body of evidence for an outcome, whether multiple reviewers worked independently, and any processes used to resolve disagreements between assessors.<br>• Report any processes used to obtain or confirm relevant information from investigators.<br>• If an automation tool was used to support the assessment of certainty, report how the automation tool was used, how the tool was trained, and details on the tool's performance and internal validation.<br>• Describe methods for reporting the results of assessments of certainty, such as the use of Summary of Findings tables.<br>• If standard phrases that incorporate the certainty of evidence were used (e.g. "hip protectors probably reduce the risk of hip fracture slightly"), report the intended interpretation of each phrase and the reference for the source guidance.	• The Grading of Recommendations Assessment, Development, and Evaluation (GRADE) [17] system was used to evaluate the quality of evidence.<br>• according to the following parameters: risk of bias, inconsistency, indirectness, imprecision, and publication bias.<br>o Not applicable<br>• The quality of the evidence was classified into four levels (high, moderate, low, and very low)<br>• This assessment was assessed by two indepedent authors (JCC and CKM). Any disagreement was solved by consensus<br>o Not applicable<br>o Not applicable<br>•<br>•

RESULTS			
STUDY SELECTION (flow of studies)	16a	• Report, ideally using a flow diagram, the number of: records identified; records excluded before screening; records screened; records excluded after screening titles or titles and abstracts; reports retrieved for detailed evaluation; potentially eligible reports that were not retrievable; retrieved reports that did not meet inclusion criteria and the primary reasons for exclusion; and the number of studies and reports included in the review. If applicable, also report the number of ongoing studies and associated reports identified.<br>• If the review is an update of a previous review, report results of the search and selection process for the current review and specify the number of studies included in the previous review.<br>• If applicable, indicate in the PRISMA flow diagram how many records were excluded by a human and how many by automation tools.	• Figure 1 shows the PRISMA flowchart for the summarized results. The main characteristics of each study are summarized in Table 1.<br>• Not applicable<br>• Not applicable
STUDY SELECTION (excluded studies)	16b	• Cite studies that might appear to meet the inclusion criteria, but which were excluded, and explain why they were excluded.	• Table 2 shows the characteristics of the studies not included in the meta-analysis including the reasons for exclusion.

Section and Topic	Item #	Elements recommended for reporting	
STUDY CHARACTERISTICS	17	• Cite each included study.<br>• Present the key characteristics of each study in a table or figure (considering a format that will facilitate comparison of characteristics across the studies).<br>• If the review examines the effects of interventions, consider presenting an additional table that summarises the intervention details for each study.	• Paragraph 1<br>• Table 1<br>• Not applicable
RISK OF BIAS IN STUDIES	18	• Present tables or figures indicating for each study the risk of bias in each domain/component/item assessed (e.g. blinding of outcome assessors, missing outcome data) and overall study-level risk of bias.<br>• Present justification for each risk of bias judgement, for example in the form of relevant quotations from reports of included studies.<br>• If assessments of risk of bias were done for specific outcomes or results in each study, consider displaying risk of bias judgements on a forest plot, next to the study results.	• Not applicable
RESULTS OF INDIVIDUAL STUDIES	19	• For all outcomes, irrespective of whether statistical synthesis was undertaken, present for each study summary statistics for each group (where appropriate). For dichotomous outcomes, report the number of participants with and without the events for each group; or the number with the event and the total for each group (e.g. 12/45). For continuous outcomes, report the mean, standard deviation and sample size of each group.<br>• For all outcomes, irrespective of whether statistical synthesis was undertaken, present for each study an effect estimate and its precision (e.g. standard error or 95% confidence/credible interval). For example, for time-to-event outcomes, present a hazard ratio and its confidence interval.<br>• If study-level data is presented visually or reported in the text (or both), also present a tabular display of the results.<br>• If results were obtained from multiple data sources (e.g. journal article, study register entry, clinical study report, correspondence with authors), report the source of the data.	• Table 1<br>• Not applicable<br>• Not applicable<br>• Not applicable<br>•

		• If applicable, indicate which results were not reported directly and had to be computed or estimated from other information.	
RESULTS OF SYNTHESES (characteristics of contributing studies)	20a	• Provide a brief summary of the characteristics and risk of bias among studies contributing to each synthesis (meta-analysis or other). The summary should focus only on study characteristics that help in interpreting the results (especially those that suggest the evidence addresses only a restricted part of the review question, or indirectly addresses the question).<br>• Indicate which studies were included in each synthesis (e.g. by listing each study in a forest plot or table or citing studies in the text).	• Risk of bias section<br>• Sup file 3
RESULTS OF SYNTHESES (results of statistical syntheses)	20b	• Report results of all statistical syntheses described in the protocol and all syntheses conducted that were not pre-specified.<br>• If meta-analysis was conducted, report for each: o the summary estimate and its precision (e.g. standard error or 95% confidence/credible interval) o measures of statistical heterogeneity (e.g. τ , I^2, prediction interval)<br>• If other statistical synthesis methods were used (e.g. summarising effect estimates, combining P values), report the synthesized result and a measure of precision (or equivalent information, for example, the number of studies and total sample size).<br>• If the statistical synthesis method does not yield an estimate of effect (e.g. as is the case when P values are combined), report the relevant statistics (e.g. P value from the statistical test), along with an interpretation of the result that is consistent with the question addressed by the synthesis method.<br>• If comparing groups, describe the direction of effect (e.g. fewer events in the intervention group, or higher pain in the comparator group).<br>• If synthesising mean differences, specify for each synthesis, where applicable, the unit of measurement (e.g. kilograms or pounds for weight), the upper and lower limits of the measurement scale (e.g. anchors range from 0 to 10), direction of benefit (e.g. higher scores denote higher severity of pain), and the minimally important difference, if known. If synthesising standardised mean differences, and the effect estimate is being re-expressed to a particular instrument, specify details of the instrument, as per the mean difference.	• Results section<br>• Results section<br>• Not applicable<br>• Results section<br>• Results section
RESULTS OF SYNTHESES (results of investigations of heterogeneity)	20c	• If investigations of possible causes of heterogeneity were conducted: o present results regardless of the statistical significance, magnitude, or direction of effect modification.<br>o identify the studies contributing to each subgroup.	• Results section

		o report results with due consideration to the observational nature of the analysis and risk of confounding due to other factors.<br>• If subgroup analysis was conducted:	

Section and Topic	Item #	Elements recommended for reporting	
		o report for each analysis the exact P value for a test for interaction, as well as, within each subgroup, the summary estimates, their precision (e.g. standard error or 95% confidence/credible interval) and measures of heterogeneity.<br>o consider presenting the estimate for the difference between subgroups and its precision.<br>• If meta-regression was conducted: o report for each analysis the exact P value for the regression coefficient and its precision.<br>o consider presenting a meta-regression scatterplot with the study effect estimates plotted against the potential effect modifier.<br>• If informal methods (i.e. those that do not involve a formal statistical test) were used to investigate heterogeneity, describe the results observed.	o
RESULTS OF SYNTHESES (results of sensitivity analyses)	20d	• If any sensitivity analyses were conducted: o report the results for each sensitivity analysis. o comment on how robust the main analysis was given the results of all corresponding sensitivity analyses.<br>o consider presenting results in tables that indicate: (i) the summary effect estimate, a measure of precision (and potentially other relevant statistics, for example, I^2 statistic) and contributing studies for the original meta-analysis; (ii) the same information for the sensitivity analysis; and (iii) details of the original and sensitivity analysis assumptions.<br>o consider presenting results of sensitivity analyses visually using forest plots.	Results section

REPORTING BIASES	21	• Present assessments of risk of bias due to missing results (arising from reporting biases) for each synthesis assessed.<br>• If a tool was used to assess risk of bias due to missing results in a synthesis, present responses to questions in the tool, judgements about risk of bias and any information used to support such judgements.<br>• If a funnel plot was generated to evaluate small-study effects (one cause of which is reporting biases), present the plot and specify the effect estimate and measure of precision used in the plot. If a contour-enhanced funnel plot was generated, specify the 'milestones' of statistical significance that the plotted contour lines represent (P = 0.01, 0.05, 0.1, etc.)<br>• If a test for funnel plot asymmetry was used, report the exact P value observed for the test, and potentially other relevant statistics, for example the standardised normal deviate, from which the P value is derived.<br>• If any sensitivity analyses seeking to explore the potential impact of missing results on the synthesis were conducted, present results of each analysis (see item #20d), compare them with results of the primary analysis, and report results with due consideration of the limitations of the statistical method.<br>• If studies were assessed for selective non-reporting of results by comparing outcomes and analyses pre-specified in study registers, protocols, and statistical analysis plans with results that were available in study reports, consider presenting a matrix (with rows as studies and columns as syntheses) to present the availability of study results.<br>• If an assessment of selective non-reporting of results reveals that some studies are missing from the synthesis, consider displaying the studies with missing results underneath a forest plot or including a table with the available study results.	• Results section
CERTAINTY OF EVIDENCE	22	• Report the overall level of certainty (or confidence) in the body of evidence for each important outcome.<br>• Provide an explanation of reasons for rating down (or rating up) the certainty of evidence (e.g. in footnotes to an evidence summary table).<br>• Communicate certainty in the evidence wherever results are reported (i.e. abstract, evidence summary tables, results, conclusions), using a format appropriate for the section of the review.<br>• Consider including evidence summary tables, such as GRADE Summary of Findings tables.	• GRADE section
DISCUSSION			
DISCUSSION (interpretation)	23a	• Provide a general interpretation of the results in the context of other evidence.	• Paragraphy 1
DISCUSSION (limitations of evidence)	23b	• Discuss any limitations of the evidence included in the review.	• Limitation section
Section and Topic	Item #	Elements recommended for reporting	
DISCUSSION (limitations of review processes)	23c	• Discuss any limitations of the review processes used, and comment on the potential impact of each limitation.	
DISCUSSION (implications)	23d	• Discuss implications of the results for practice and policy.<br>• Make explicit recommendations for future research.	• Contributions of this study section

http://doi.org/10.47626/2237-6089-2022-0497

OTHER INFORMATION			
REGISTRATION AND PROTOCOL (registration)	24a	• Provide registration information for the review, including register name and registration number, or state that the review was not registered.	The registration was carried out in the PROSPERO database under the two following submissions: CRD42021234363 "Is medical school associated with decreased quality of life in Brazilian students? A systematic review and meta-analysis over a decade"..<br>•
REGISTRATION AND PROTOCOL (protocol)	24b	• Indicate where the review protocol can be accessed (e.g. by providing a citation, DOI or link), or state that a protocol was not prepared.	• Link
REGISTRATION AND PROTOCOL (amendments)	24c	• Report details of any amendments to information provided at registration or in the protocol, noting: (a) the amendment itself; (b) the reason for the amendment; and (c) the stage of the review process at which the amendment was implemented.	• Not applicable
SUPPORT	25	• Describe sources of financial or non-financial support for the review, specifying relevant grant ID numbers for each funder. If no specific financial or nonfinancial support was received, this should be stated.<br>• Describe the role of the funders or sponsors (or both) in the review. If funders or sponsors had no role in the review, this should be declared.	• This work was supported financially by the Conselho Nacional de Desenvolvimento Científico e Tecnológico (CNPq, Brasília, Brazil). The funding sources had no impact in the design, conduct, or reporting of the article or the decision to publish the study. The authors declare no conflict of interest.<br>•
COMPETING INTERESTS	26	• Disclose any of the authors' relationships or activities that readers could consider pertinent or to have influenced the review.<br>• If any authors had competing interests, report how they were managed for particular review processes.	• The author declare no conflict of interest
AVAILABILITY OF DATA, CODE, AND OTHER MATERIALS	27	• Report which of the following are publicly available: template data collection forms; data extracted from included studies; data used for all analyses; analytic code; any other materials used in the review.<br>• If any of the above materials are publicly available, report where they can be found (e.g. provide a link to files deposited in a public repository).<br>• If data, analytic code, or other materials will be made available upon request, provide the contact details of the author responsible for sharing the materials and describe the circumstances under which such materials will be shared.	• Not applicable<br>• Not applicable<br>• Not applicable

http://doi.org/10.47626/2237-6089-2022-0497

Supplementary Table S1 - Detailed search strategy containing the databases and the number of extracted references.

PUBMED – Search filters	***Records***
**#1 Population [Mesh]:** (Students, medical)	38,171
**#2 Outcome [Mesh]: (**quality of life)	220,359
**#3 Combined search: (**#1 AND #2)	270
**EMBASE –** ***Search filters***	***Records***
**#1 Population [tittle, abstract, keyword]:** medical student	79,660
**#2 Outcome [All fields]: (**quality of life)	560,012
**#3 Combined search: (**#1 AND #2)	176
BVS database– Search filters	***Records***
**#1 Population [title, abstract, subject]:** (estudantes de medicina)	4,197
**#2 Outcome [title, abstract, subject]: (**Qualidade de vida)	23,458
**#3 Combined search: (**#1 AND #2)	146

Supplementary Table S2 - Characteristics of studies not included in the meta-analysis (with reasons)

**Author**	**Data collection date**	**Location**	**University (abbreviation) (type of institution)**	**Population (N)**	**Percentage of enrolled population**	**QOL Evaluation instument**	**Comparisons**	**Negative predictor factors**	**Reasons for exclusion**
Paro, Morales (1)	Feb 2006 - Feb 2007	Uberlandia-MG	Universidade Federal de Uberlandia (UFU) (public)	All-students (352)	78.2%	SF-36	Academic year<br>Depression<br>Gender<br>Living arrangments	3rd year<br>Depression<br>Females	Not using the WHOQOL-bref as the instrument measure (SF-36 as the questionnaire)<br>Lack of data (scores given as median and percentiles)
Ramos-Dias, Libardi (2)	Not informed	Sorocaba-SP	Pontifício Universidade católica (PUC) (private	First and last year (100)	Not informed	WHOQOL-Bref	Academic year	First-year	Not controlling for gender
Bampi, Baraldi (3)	Aug 2010 - Aug 2011	Brasilia-DF	Universidade de Brasília (UnB) (public)	All-students (84)	18 %	WHOQOL-Bref	Questionnaire domains	Psychological domain	Not comparing the influence of the academic cycle in the QoL index
César, de Pádua Paz (4)	Aug 2009 - Nov 2009	Brasília-DF	Not informed	All-students (345)	70%	WHOQOL??	Academic year	4th year	Lack of data (standard deviation)
Chazan and Campos (5), Chazan, Campos (6)	Apr 2010 - May 2010	Rio de Janeiro – RJ	Universidade Estadual do Rio de janeiro (UERJ) (public)	All-students (394)	72 %	WHOQOL-Bref	Academic year<br>Comorbidities<br>Gender<br>Quotes<br>Social class	Third and sixth year<br>Referred chronic morbidity<br>Females<br>Quota students<br>Social class C	Lack of data (standard deviation
Meyer, Guimarães (7)	June 2011 - Sept 2011	Multicenter-SC	Multicenter (1 public; 11 private)	Last year students (302)	Not informed	WHOQOL-Bref	Gender<br>Occupational stress l(Job stress scale)	None	Not comparing the influence of the academic cycle in the QoL index
Olmo, Ferreira (8)	Not informed	Santos-SP	Universidade Metropolitana de Santos (Unimes) (private)	First and last year (108)	75 % First year<br>57 % last year	WHOQOL-Bref,	Academic year	Last-year	Not comparing the influence of the academic cycle in the QoL index
Paro and Bittencourt (9)	Not informed	Campinas-SP	Universidade Estadual de Campinas (UNICAMP) (public)	All-students (309)	47%	SF-36	Academic year	5th year	Not using the WHOQOL-bref as the instrument measure (SF-36 as the questionnaire)<br><br>Not controlling for gender
Hickel, Fabro (10)	Dec 2012 - Mar 2013	Pelotas-RS	Universidade Federal de Pelotas (UFPel) (public)	First to 7th semester	95%	WHOQOL-Bref	Academic year<br>Distance from hometown	Distance from hometown	Not comparing the last academic cycle<br>(The comparison was established between the first and 7th semester)

Lins, Carvalho (11), Lins, Carvalho (12)	Oct 2013 – Nov 2013	Salvador-BA	Escola Bahiana de Medicina e Saúde pública (EBMSP)(private)	All-students (180)	Not informed	SF-36	Academic year<br>Age (17-22/ 23-33)<br>Gender<br>Headaches,<br>Loan program<br>Living arrangements<br>Physical activity<br>Sleepines	Females,<br>Suffering from headaches<br>Lacking physical activity<br>Suffering from Sleepiness<br>Being participants of loan programs	Not using the WHOQOL-bref as the instrument measure (SF-36 as the questionnaire)<br><br>Lack of data (Not possible to address the mean and standard deviation for males and females independently)
Serinolli and El-Mafarjeh (13)	Feb 2014 - Apr 2014	São Paulo-SP	Universidade Nove de Julho (Uninove) (private)	All-students (405)	Not informed	WHOQOL-Bref	Mental diseases<br>Physical activity<br>Religiosity	Previous diagnosis of mental diseases<br>Lack of physical activity<br>Absence of religious beliefs	Not comparing the influence of academic cycle in the contr
Chagas, Sanches (14)	Oct 2015 - Dec 2015	São Carlo-SP	Universdade Federal de São Carlos (UFSCar) (public)	First to 4th year	Not informed	WHOQOL-100	Academic year	3rd year	Not academicnder
Cunha, Moraes (15)	Aug 2011 - Dec 2011	São Paulo-SP	Universidade Federal de São Paulo (UNIFESP) (public)	All-students (607)	82,5%	WHOQOL-100	Academic cycle<br>Academic year<br>Gender<br>Social class	6th year<br>Females<br>Social class C/D	Not controlling for gender
Pereira, Ribeiro (16)	Not informed	Patos-PB	Faculdade integrada de Patos (FIP) – (private)	First to the fifth semester (138)	76,7%	WHOQOL-Bref	Academic semester<br>Age<br>Burnout<br>Gender<br>Loan programs<br>Mental disorder<br>Physical activity<br>Satisfaction with the course<br>Stress	Females<br>Burnout<br>Presence of mental disorders<br>Satisfaction with the course<br>Stress	Lack of data
dos Santos, Ribeiro (17)	2016	Jequié-BA	Universidade Estadual do Sudoeste da Bahia (UESB) (public)	Not informed	Not informed	WHOQOL-Bref	Common Mental Disorders	Presence of psychiatric syntoms	Not comparing the influence of the academic cycle in the QoL index
Antunes, Silva Menezes (18)	2014	Salvador-BA	Escola Bahiana de Medicina e Saúde pública (EBMSP)(private)	All-students (291)	Not informed	VERAS-Q	Academic cycle<br>Gender	Last academic cycle<br>Females	WHOQOL-bref as the instrument measure (VERAS-Q as the questionnaire)<br><br>Not controlling for sex
Cavalcante, Cazolari (19)	Aug 2017 - Dec 2018	São Paulo-SP	Universidade Federal de São Paulo (UNIFESP)	First and last-year	Not informed	WHOQOL-Bref	Academic year	Last-year	Not controlling for gender

			(public)						
Andrade, Caetano (20)	June 2018 – not informed	Franca-SP	Universidade de Franca (UNIFRAN) (private)	First to 4th year (n=310)	Not informed	WHOQOL-Bref	Academic year<br>Gender<br>Studying hours	First-year<br>Females	Not comparing the last academic cycle<br>(The comparison was established between the first to the 4th year)
Durán and Dunningham (21)	Not informed	Salvador-BA	Centro Universitário Faculdades de Tecnologia e Ciências (UniFTC) (private)	First to 8th semester (80)	Not informed	SF-36	Workload	High workloads	Not using the WHOQOL-bref as the instrument measure (SF-36 as the questionnaire)<br><br>Not controlling for gender
Meyer, Barbosa (22)	Not informed	Multicenter-SC	Multicenter (1 public; 8 private)	Last year students (508)	Not informed	WHOQOL-Bref	The difficulty of conciliation of clerkship and studies	The difficulty of conciliating clerkship and studies	Not comparing the influence of the academic cycle in the QoL index
Moutinho, Lucchetti (23)	From 2014 to 2016 (4 semesters follow up)	Juiz de Fora-MG	Universidade Federal de Juiz de fora (UFJF) (public)	Students were included if they could be followed for two years (312)	54.2%	WHOQOL-Bref	Anxiety<br>Depression<br>Ethnicity<br>Gender<br>Religiosity<br>Social class<br>Stress	Anxiety<br>Depression,<br>Non-white students<br>females<br>Having low income,<br>High-stress levels	Not comparing the last academic cycle<br>(The comparison was established between the first to the 4th semester)
Rocha 2019	Jan 2018 - June 2018	Franca-SP	Universidade de Franca (UNIFRAN) (private)	All-students	Not informed	WHOQOL-Bref<br>VERAS	Academic year	3rd and 5th year	Not comparing the last academic cycle<br>(The comparison was established between the 3rd and 5th year)
Cangussu, Ezequiel (24)	2015	Juiz de fora-MG	Universidade Federal de Juiz de Fora (UFJF) (public)	All-students (776)	77%	WHOQOL-Bref	Academic cycle<br>Gender	First cycle<br>Females	Lack of data (the authors gave the standard error instead of the standard deviation and the total number of participants was not available for each group)
Miranda, Tavares (25)	Not informed	Aparecida de goiânia-GO	Universidade de Rio Verde (UniRV) (private)	Second to 8th semester (419)	Not informed	WHOQOL-Bref	Comorbidities<br>Food consumption<br>Gender<br>Practicing physical activity<br>Stimulants use<br>Thinking about giving up medical school	Presence of comorbidities.<br>females<br>Use of stimulants,<br>Thinking about giving up medical school	Not comparing the first academic cycle in the QoL index
Pires, Gusmão (26)	Aug 2017 - Dec 2017	Maceió-AL	Universidade Estadual de Ciências da Saúde	All-students (190)	63%	WHOQOL-Bref	Academic cycle<br>Gender	Last academic cycle<br>Females	Lack of data (the total number of participants was not available for each sex group)

			de Alagoas (Uncisal) (public)						
Silva, Pereira (27)	Not informed	Caratinga-MG	Centro universitário de Caratinga (UNEC) (private)	First, third and fifth year (n=94)	64% First year<br>50% Second year<br>51,1% Fifth year	WHOQOL-Bref	Minor mental disorders	Prevalence of minor mental disorders	Not comparing the influence of the academic cycle in the QoL index

References

1. Paro HB, Morales NM, Silva CH, Rezende CH, Pinto RM, Morales RR, et al. Health-related quality of life of medical students. Medical education. 2010;44(3):227-35.
2. Ramos-Dias JC, Libardi MC, Zillo CM, Igarashi MH, Senger MH. Qualidade de vida em cem alunos do curso de Medicina de Sorocaba-PUC/SP. Revista Brasileira de Educação Médica. 2010;34:116-23.
3. Bampi LNdS, Baraldi S, Guilhem D, Araújo MPd, Campos ACdO. Qualidade de vida de estudantes de Medicina da Universidade de Brasília. Revista Brasileira de Educação Médica. 2013;37:217-25.
4. César BN, de Pádua Paz I, Novaes MRCG. Aplicação do instrumento de avaliação da qualidade de vida do estudante de medicina em escola pública de Brasília. Revista de Ciências Médicas. 2012;21(1/6):79-86.
5. Chazan ACS, Campos MR. Qualidade de vida de estudantes de medicina medida pelo WHOQOL-bref-UERJ, 2010. Revista brasileira de educação médica. 2013;37:376-84.
6. Chazan ACS, Campos MR, Portugal FB. Qualidade de vida de estudantes de medicina da UERJ por meio do Whoqol-bref: uma abordagem multivariada. Ciência Saúde Coletiva. 2015;20:547-56.
7. Meyer C, Guimarães ACdA, Machado Z, Parcias SR. Qualidade de vida e estresse ocupacional em estudantes de medicina. Revista brasileira de educação médica. 2012;36:489-98.
8. Olmo NRS, Ferreira LF, Prado AD, Martins LC, Dedivitis RA. Percepção dos estudantes de medicina do primeiro e sexto anos quanto à qualidade de vida. Diagn tratamento. 2012;17(4):157-61.
9. Paro CA, Bittencourt ZZLdC. Qualidade de vida de graduandos da área da saúde. Revista brasileira de educação médica. 2013;37:365-75.
10. Hickel F, Fabro BR, Bertoldi EG. Fatores de risco cardiovascular e qualidade de vida de acadêmicos de medicina da Universidade Federal de Pelotas. Rev AMRIGS. 2015;1:186-91.
11. Lins L, Carvalho FM, Menezes MS, Porto-Silva L, Damasceno H. Health-related quality of life of students from a private medical school in Brazil. International journal of medical education. 2015;6:149-54.
12. Lins L, Carvalho FM, Menezes MS, Porto-Silva L, Damasceno H. Health-related quality of life of medical students in a Brazilian student loan programme. Perspect Med Educ. 2016;5(4):197-204.
13. Serinolli MI, El-Mafarjeh E. Impacto da prática de atividade física na qualidade de vida dos acadêmicos de Medicina da Universidade Nove de Julho (Uninove). ConScientiae Saúde. 2015;14(4):627-33.

14. Chagas NB, Sanches FB, Silva RFd, Melo DG, Germano CMR, Avó LRdS. Qualidade de Vida de Estudantes de Medicina em um Curso que Adota Metodologias Ativas de Ensino-Aprendizagem. Revista brasileira de educação médica. 2018;42:96-102.
15. Cunha DHFd, Moraes MAd, Benjamin MR, Santos AMN. Percepção da qualidade de vida e fatores associados aos escores de qualidade de vida de alunos de uma escola de medicina. Jornal Brasileiro de Psiquiatria. 2017;66:189-96.
16. Pereira F, Ribeiro C, Oliveira L, Araujo Filho J, Tabosa M, Gouveia Filho P, et al. Correlatos da qualidade de vida com características de saúde e demográficas de estudantes de medicina. Rev Bras Qual Vida. 2017;9:247-60.
17. dos Santos LS, Ribeiro ÍJS, Boery EN, de Oliveira Boery RNS. Qualidade de vida e transtornos mentais comuns em estudantes de medicina. Cogitare Enfermagem. 2017;22(4).
18. Antunes BR, Silva Menezes M, Lins L. Quality of life of medical students in Brazil: a comparative study. Rev méd Chile. 2019:107-13.
19. Cavalcante MS, Cazolari PG, Galliano SA, Cohrs FM, Sañudo A, Schveitzer MC. Qualidade de vida dos estudantes do primeiro e sexto ano do curso de Medicina. Revista de Medicina. 2019;98(2):99-107.
20. Andrade FKd, Caetano LAO, Oliveira WAd, Silva JLd, Manochio-Pina MG. Qualidade de vida e burnout entre estudantes de medicina que vivenciam o mÚtodo de Aprendizagem Baseada em Problemas. Aletheia. 2019;52(1):116-28.
21. Durán FC, Dunningham WA. Relação entre a carga horária e a qualidade de vida dos alunos do curso de medicina de uma faculdade de salvador. Revista Brasileira de Neurologia e Psiquiatria. 2019;23(3).
22. Meyer C, Barbosa DG, Andrade RD, Junior GJF, Neto MGF, Guimarães AC, et al. Qualidade de vida de estudantes de medicina e a dificuldade de conciliação do internato com os estudos. ABCS Health Sciences. 2019;44(2).
23. Moutinho ILD, Lucchetti ALG, Ezequiel ODS, Lucchetti G. Mental health and quality of life of Brazilian medical students: Incidence, prevalence, and associated factors within two years of follow-up. Psychiatry research. 2019;274:306-12.
24. Cangussu SA, Ezequiel OS, Lucchetti ALG, DiLalla LF, Lucchetti G. Empathy, well-being, and mental health: do gender differences diminish by the end of medical school? Women & Health. 2021;61(3):254-64.
25. Miranda IMM, Tavares HHF, Silva HRSd, Braga MS, Santos RdO, Guerra HS. Qualidade de Vida e Graduação em Medicina. Revista Brasileira de Educação Médica. 2020;44.
26. Pires AMFdS, Gusmão WDP, Carvalho LWTd, Amaral MMLdS. Qualidade de Vida de Acadêmicos de Medicina: Há Mudanças durante a Graduação? Revista Brasileira de Educação Médica. 2020;44.
27. Silva RC, Pereira AdA, Moura EP. Qualidade de Vida e Transtornos Mentais Menores dos Estudantes de Medicina do Centro Universitário de Caratinga (UNEC)-Minas Gerais. Revista Brasileira de Educação Médica. 2020;44.
